# Expression of RET is associated with Oestrogen receptor expression but lacks prognostic significance in breast cancer

**DOI:** 10.1186/s12885-018-5262-0

**Published:** 2019-01-08

**Authors:** Robert Mechera, Savas D. Soysal, Salvatore Piscuoglio, Charlotte K. Y. Ng, Jasmin Zeindler, Edin Mujagic, Silvio Däster, Philippe Glauser, Henry Hoffmann, Ergin Kilic, Raoul A. Droeser, Walter P. Weber, Simone Muenst

**Affiliations:** 1 Department of Surgery, University Hospital Basel, Spitalstrasse 21, 4031 Basel, Switzerland; 2 Institute of Pathology, University Hospital Basel, Schoenbeinstrasse 40, 4031 Basel, Switzerland; 3 Institute of Pathology, Klinikum Leverkusen, Am Gesundheitspark 11, 51375 Leverkusen, Germany

**Keywords:** RET, Breast cancer, Oestrogen receptor, Endocrine resistance, Tissue microarray

## Abstract

**Background:**

The Rearranged during Transfection (RET) protein is overexpressed in a subset of Estrogen Receptor (ER) positive breast cancer, with both signalling pathways functionally interacting. This cross-talk plays a pivotal role in the resistance of breast cancer cells to anti-endocrine therapies, and RET expression is assumed to correlate with poor prognosis based on findings in small patient cohorts. The aim of our study was to investigate the impact of RET expression on patient outcome in human breast cancer.

**Methods:**

We performed an immunohistochemical analysis of RET protein expression on a tissue microarray encompassing 990 breast cancer patients and correlated its expression with clinicopathological parameters and survival data.

**Results:**

Expression of RET was detected in 409 out of 990 cases (41.3%). RET and ER expression significantly correlated (*p* < 0.0001). The Luminal B HER2-positive subtype showed the highest expression rate (48.9%). In univariate and multivariate survival analyses, RET expression had no impact on overall survival.

**Conclusion:**

We confirmed the co-expression of RET and ER, but we did not find RET expression to be an independent prognostic factor in human breast cancer. Clinical trials with newly developed RET inhibitors are needed to evaluate if RET inhibition has a beneficial impact on patient survival in ER positive breast cancer.

## Background

The Rearranged during Transfection (RET) protein belongs to the receptor tyrosine kinase (RTK) superfamily encoded by the *RET* gene on the human chromosome 10q11.2 [1]. Ligands of the E glial cell line-derived neurotrophic factor (GDNF) family of growth factors bind to one of four GDNF family α-receptors (GFRα1–4) leading to RET dimerization and trans-phosphorylation of intracellular tyrosines [2], which in turn regulate cellular differentiation, survival, proliferation, migration and chemotaxis [1]. The first causative oncogenic role of the *RET* gene was identified in human papillary thyroid carcinoma (PTC) [3–5]. Moreover, *RET* seems to also play a role in many other cancer entities and cancer syndromes [6–15].

The concept of activated receptor protein kinases in breast cancer has been well established, as overexpression or amplification promotes tumour growth [1, 7–10]. With regard to RET, results have been initially conflicting [11, 12]. However, RET has been increasingly gaining attention [13], and overexpression of *RET* and its coreceptor *GFRα1* was demonstrated in a subset of hormone receptor positive breast cancers [4]. Moreover, a functional interaction between the RET and ER signalling pathways has been shown in breast cancer cell line studies [6, 14]: First, oestrogen stimulation seems to highly upregulate *RET* and *GFRα* mRNA, suggesting that *RET* and *GFRα* are direct target genes of oestrogen signalling [6]. Secondly, RET activation has been demonstrated to increase ER*α* phosphorylation as well as ER-independent transcriptional activation of ER*α* target genes [14], leading to an increased oncogenicity and potentiation of oestrogen-driven proliferation [6].

The molecular mechanisms involved in the cross-talk between upstream kinases and ER*α* play a pivotal role in the resistance to anti-endocrine therapies [1, 14, 15] and RET expression seems to be associated with disease recurrence after adjuvant Tamoxifen treatment [14]. An additional mechanism of RET in endocrine resistance is the interaction with inflammatory cytokines. RET expression increases interleukin (IL)-6 levels in the presence of endocrine treatment, resulting in a positive-feed forward loop [16]. Due to the important role of IL-6 in breast cancer cell migration, RET not only seems to have an impact on tumour growth but also on metastasis [16, 17].

Supported by the association of RET expression and poor prognosis [15–17] as well as an association with negative prognostic factors such as large tumour size [16], the combination of endocrine therapy with agents blocking the RET signalling pathway could be a possible approach to overcome endocrine resistance in breast cancer, and has become the subject of preclinical research and various clinical trials [1, 15, 18, 19]. Several preclinical studies have shown at least a partial reversibility of endocrine resistance in vivo and in vitro with RET inhibitors [15, 18–20]. However, early clinical trials using RET inhibitors alone or in combination with aromatase inhibitors have struggled with either high toxicity or lack of benefit [1].

In summary, due to its specific role in endocrine resistance as well as the possible correlation with poor prognosis, RET remains a promising therapeutic target in breast cancer [16]. However, analyses of RET expression and association with clinicopathological parameters including survival data in larger patient cohorts are missing. To further investigate the role of RET expression in human breast cancer we performed an immunohistochemical analysis on breast cancer tissue microarrays (TMA) with detailed clinical and survival data. This study is reported according to the reporting recommendations for tumour marker prognostic studies (REMARK) [21].

## Material and methods

### Tissue microarray

Six Tissue Microarrays (TMA) encompassing a total of 1624 breast cancer tissue punches originating from formalin-fixed and paraffin-embedded tumour tissue were used and assembled into a TMA format as previously described [22, 23]. The specimens derived from patients diagnosed with primary breast cancer between 1985 and 2015 (approximately 90% of them between 1985 and 1995) at the Institute of Pathology and the private Institute Boss and Spichtin, Switzerland. Due to loss of tissue on individual punches, a total of 990 samples could be evaluated. The loss of tissue is explained by the fact that the TMA have already been used multiple times for various scientific projects and therefore some of the punches are depleted. Additionally, not all tissue punches do indeed contain cancer tissue, but rather benign surrounding tissue, which could not be included in our analysis. Histopathological data was obtained from the individual pathology reports while clinical and survival data were extracted from the hospital database, Cancer Registry of Basel or from the patients’ attending physicians. Ethical standards and patients’ confidentiality were ensured and in line with regulations of the local institutional review board (Ethikkomission Nordwest- und Zentralschweiz, EKNZ 2014–397) and data safety laws.

### Immunohistochemistry

For immunohistochemical staining, 4 μm sections of the TMA blocks were incubated for 12 min with the polyclonal membranous and cytoplasmatic anti-RET antibody (Clone ab133710, Abcam, Cambridge, UK) in a dilution of 1:50 after heat induced antigen retrieval with citrate buffer at pH 6 for 16 min. Standard-technique for Benchmark Ultra with optiView system was employed. Counterstaining was performed with hematoxylin solution. For Ki-67 (Clone IR626, Dako, Santa Clara, CA, USA) immunostaining was performed on Benchmark Ultra with optiView system. ER, Progesterone Receptor (PR) and HER2 were stained and scored as previously described [24]. For 88% of the breast cancer cases, ER, PR and HER2 were evaluated on the TMA punches, for the other 12%, these markers were evaluated on whole slide sections of the donor blocks.

**Fig. 1:**
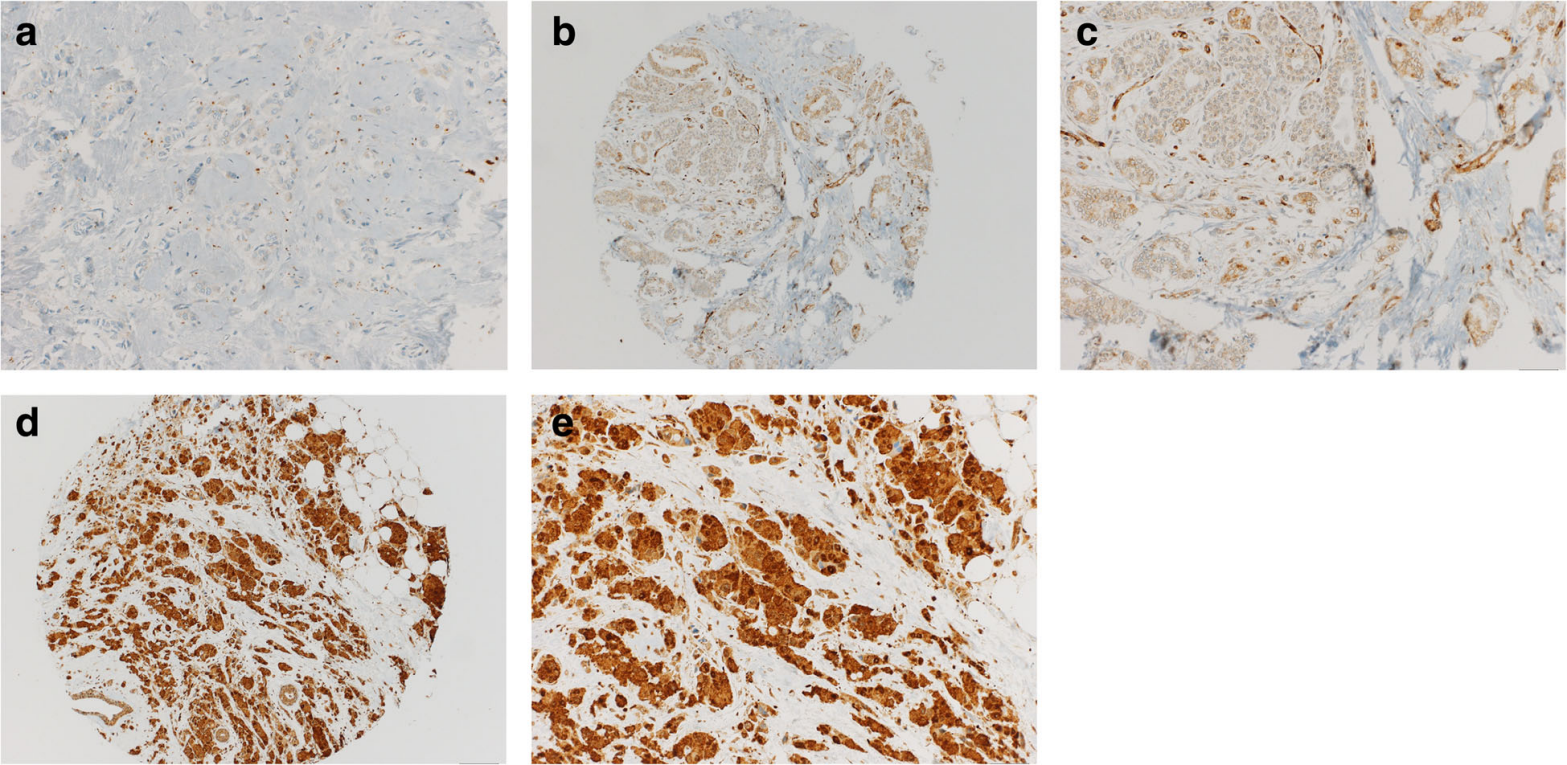
Five representative photographs of breast cancer tissue punches with immunohistochemical staining of breast cancer cells for RET **a**: absent staining (0), Magnification 200×; **b**: weak intensity (1+), Magnification 100×; **c**: weak intensity (1+), Magnification 200×; **d**: strong intensity (3+), Magnification 100×; **e**: strong intensity (3+), Magnification 200×

The analysis of RET expression was performed by two observers (RM and SM), both blinded to the histopathological, clinical and survival data. The RET expression was scored as [24]: 0 = absent staining, 1 = weak intensity, 2 = intermediate intensity, 3 = strong intensity (Fig. 1). For the statistical analysis, the score was dichotomised into absent staining and weak intensity (RET negative) versus intermediate and strong intensity (RET positive). Since the staining was evenly distributed across all tumour cells per cancer sample, the proportion of stained tumour cells was not assessed.

### Statistical analysis

Statistical analyses for categorical and non-categorical variables were performed using Fisher’s Exact and -Chi-squared tests, respectively. Overall survival was calculated using the Kaplan-Meier method and differences between groups assessed using log-rank tests. Univariate and multivariate analyses for overall survival on clinicopathologic parameters and RET expression were performed using the Cox proportional hazards regression model. Hazard ratios and corresponding 95% confidence intervals were estimated. All tests were two-sided. *P*-values < 0.05 were considered statistically significant. All analyses were performed using Graphpad Prism 6.0 (Graphpad Software, Inc., La Jolla, CA), SPSS version 20 (IBM, Armonk, NY) or R v3.4.2.

## Results

The mean age of all 990 evaluable patients was 64 years (SD +/− 14) at the time of diagnosis and mean follow up time was 80.8 months (range 1–263). Most of the tumours (78.4%) were less than 5 cm in diameter (T1-T2), without lymph node involvement at the time of diagnosis (51.3%) and could be allocated to the intrinsic luminal B subtype (53.0%).

The relatively high proportion of luminal B subtype cancers in our population could be partly due to the low threshold of Ki-67at ≥14% and the fact that Ki-67 was evaluated on the TMA tissue punches, which does not account for heterogeneity of Ki-67 distribution and might have led to an overestimation of Ki-67 expressing cells in some cancers. Other than that, there is no obvious explanation for the high incidence of luminal B subtypes.

Detailed demographic information of the patients can be found in Table 1.

**Table 1:** Basic demographic data of all evaluable breast cancer cases (*n* = 990)

Mean tumour size (mm) ± SD	32 ± 18	
Mean age at diagnosis (years) ± SD	64 ± 14	
	Number	Percentage
	(n)	(%)
Tumour stage		
pT1	236	23.8
pT2	541	54.6
pT3	85	8.6
pT4	127	12.8
NA	1	0.2
Lymph node involvement		
pN0	508	51.3
pN1	370	37.4
pN2	98	9.9
pN3	8	0.8
NA	6	0.6
Tumour grade		
1	206	20.8
2	354	35.8
3	429	43.3
NA	1	0.1
Histologic subtype		
Invasive ductal	739	74.6
Invasive lobular	107	10.8
Mucinous	25	2.5
Apocrine	19	1.9
Cribriform	32	3.2
Others	65	6.6
NA	3	0.4
Intrinsic subtype		
Luminal A-like *(ER*^*+*^*and/or PR*^*+*^*, HER2*^*−*^*, Ki-67 < 14%)*	124	12.5
Luminal B-like (HER2-negative) *(ER*^*+*^*and/or PR*^*+*^*, HER2*^*−*^*, Ki-67 ≥ 14%)*	435	43.9
Luminal B-like (HER2-positive) *(ER*^*+*^*and/or PR*^*+*^*, HER2*^*+*^*)*	90	9.1
HER2 type *(ER*^*−*^, *PR*^*−*^*, HER2*^*+*^*)*	77	7.8
Basal-like *(ER*^*−*^*, PR*^*−*^*, HER2*^*−*^*)*	260	26.3
NA	4	0.4

The expression of RET was significantly associated with the expression of ER (*p* < 0.0001, Table 2). No significant association of RET expression with tumour size, patient’s age, tumour grade and AJCC primary tumour staging system (TNM) such as tumour size (pT) and lymph node involvement (pN) was identified. Moreover, no significant association was found between RET expression and the expression of HER2 as well as the proliferation marker Ki-67 (Table 2). Looking at the breast cancer intrinsic subtypes as defined by the St. Gallen consensus conference [25], RET expression differed significantly between the five subtypes. The lowest expression was observed in basal-like subtype (31.2%) followed by HER2 type (36.4%), luminal B subtype (HER2-negative) (45.3%) and luminal A subtype (46.0%). Of all intrinsic subtypes, the luminal B HER2-positive subtype showed the highest expression rate (48.9%, *p* = 0.001, Table 3). In summary, ER expressing subtypes show a RET expression in 45–46% of cases, while ER-negative subtypes express RET in only 31–36% of cases.

**Table 2:** Association between RET expression and clinicopathological parameters

Clinicopathologic parameter	RET-positive		RET-negative		*p* value
Mean tumour size (mm) ± SD	31 ± 17		32 ± 18		0.309
Mean age at diagnosis (years) ± SD	64 ± 14		64 ± 14		0.550
	(n)	(%)	(n)	(%)	
Tumour stage					0.546
pT1	99	41.9	137	58.1	
pT2	224	41.4	317	58.6	
pT3	39	45.9	46	54.1	
pT4	46	36.2	81	63.8	
NA	1	100	0	0	
Lymph node involvement					0.468
pN0	201	39.6	307	60.4	
pN1	163	44.1	207	55.9	
pN2	37	37.8	61	62.2	
pN3	4	50.0	4	50.0	
NA	4	66.7	2	33.3	
Tumour grade					0.401
1	91	44.2	115	55.8	
2	137	38.7	217	61.3	
3	181	42.2	248	57.8	
NA	0	0	1	100	
Oestrogen receptor					< 0.0001
ER^+^	295	46.0	347	54.0	
ER^−^	114	32.8	234	67.2	
HER2					0.666
HER2^+^	72	43.1	95	56.9	
HER2^−^	337	40.9	486	59.1	
Ki67					0.635
Ki67^-high^	331	41.7	463	58.3	
Ki67^-low^	75	39.5	115	60.5	
NA	3	50.0	3	50.0

**Table 3:** Association between RET expression and breast cancer intrinsic subtype

Intrinsic subtype	RET-positive		RET-negative		*p* value
	(n)	(%)	(n)	(%)	0.001
Luminal A *(ER*^*+*^*and/or PR*^*+*^*, HER2*^*−*^*, Ki-67 < 14%)*	57	46.0	67	54.0	
Luminal B (HER2-negative) *(ER*^*+*^*and/or PR*^*+*^*, HER2*^*−*^*, Ki-67 ≥ 14%)*	197	45.3	238	54.7	
Luminal B (HER2-positive) *(ER*^*+*^*and/or PR*^*+*^*, HER2*^*+*^*)*	44	48.9	46	51.1	
HER2 type *(ER*^*−*^*, PR*^*−*^*, HER2*^*+*^*)*	28	36.4	49	63.6	
Basal-like *(ER*^*−*^*, PR*^*−*^*, HER2*^*−*^*)*	81	31.2	179	68.8	
NA	2	50.0	2	50.0	

**Fig. 2:**
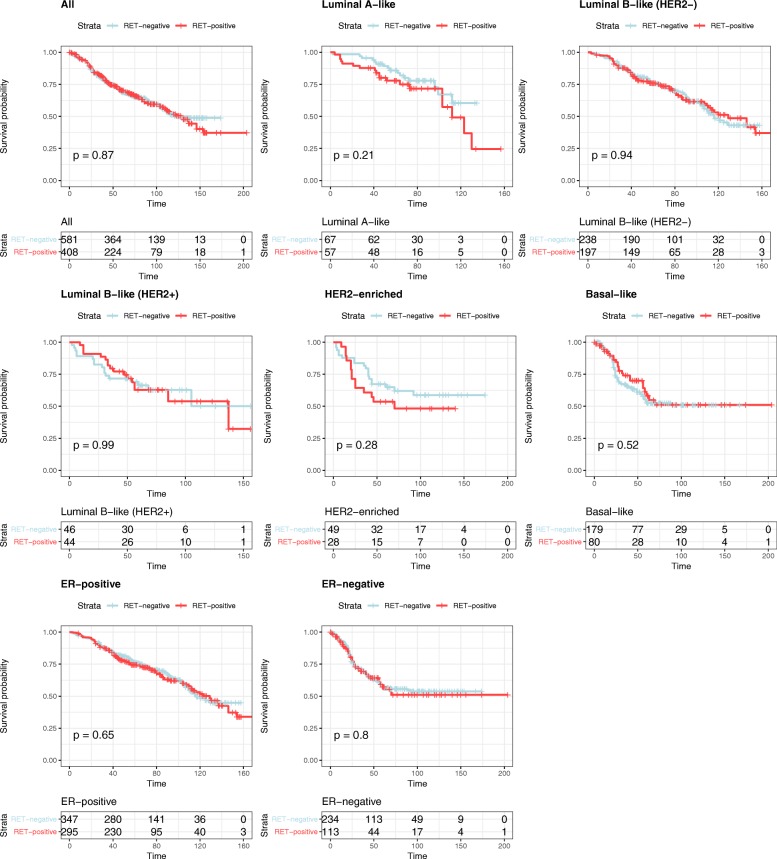
**a**: Kaplan–Meier survival curve for overall survival depending on the expression of RET (univariate analysis); **b–f**: Kaplan–Meier survival curves for overall survival depending on the expression of RET for the indicated breast cancer intrinsic subtypes

In the univariate survival analysis, RET expression had no significant impact on overall survival (OS) of breast cancer patients (*p* = 0.87) In particular, subgroup analysis of intrinsic subtypes revealed no correlation of RET expression with overall survival (Table 4, Fig. 1). For multivariate analysis, we adjusted for grade, tumour size (pT) and lymph node status (pN), age and intrinsic subtypes. We were able to confirm that age (*p* < 0.0001), tumour size (pT) (*p* < 0.0001), lymph node involvement (pN) (*p* < 0.0001) and tumour grade (*p* < 0.0001) were independent prognostic factors and correlated with worse overall survival. Importantly, RET expression had no significant impact on overall survival and is thus not a prognostic factor in human breast cancer in our collective (*p* = 0.79, Table 5, Fig. 2).

**Table 4:** Univariate analyses for all cases, and by intrinsic subtype, for the effect of RET expression on overall survival

RET expression, all cases	Hazard Ratio (95% CI)	*p* value
RET positivity	1.02 (0.82–1.26)	0.87
RET positivity, by intrinsic subtype		
Luminal A *(ER*^*+*^*and/or PR*^*+*^*, HER2*^*−*^*, Ki-67 < 14%)*	1.54 (0.79–3.00)	0.21
Luminal B (HER2-negative) *(ER*^*+*^*and/or PR*^*+*^*, HER2*^*−*^*, Ki-67 ≥ 14%)*	0.99 (0.72–1.36)	0.95
Luminal B (HER2-positive) *(ER*^*+*^*and/or PR*^*+*^*, HER2*^*+*^*)*	1.00 (0.52–1.95)	0.99
HER2 type *(ER*^*−*^*, PR*^*−*^*, HER2*^*+*^*)*	1.46 (0.73–2.92)	0.28
Basal-like *(ER*^*−*^*, PR*^*−*^*, HER2*^*−*^*)*	0.86 (0.54–1.37)	0.52
RET positivity, by ER status		
ER^+^	1.06 (0.82–1.38)	0.65
ER^−^	1.05 (0.72–1.52)	0.81

**Table 5:** Multivariate analysis for the effect of clinicopathologic parameters and RET expression on overall survival

Clinicopathologic parameter	Hazard Ratio (95% CI)	*p*-value
Age (per 1-year)	1.04 (1.03–1.05)	< 0.0001
Tumour size (pT)	1.29 (1.14–1.45)	< 0.0001
Lymph node involvement (pN)	1.47 (1.25–1.73)	< 0.0001
Tumour grade (BRE)	1.77 (1.50–2.10)	< 0.0001
Intrinsic subtype		
Luminal A	1	
Luminal B (HER2-negative)	0.96 (0.66–1.39)	0.81
Luminal B (HER2-positive)	1.03 (0.64–1.65)	0.92
HER 2 type	0.86 (0.51–1.45)	0.58
Basal-like	1.43 (0.94–2.18)	0.09
RET expression		
RET-positive	1.03 (0.83–1.28)	0.79

## Discussion

In the present study, we analysed the role of RET expression in a large cohort of 990 primary breast cancer cases and correlated the expression with clinicopathological parameters and survival data. We were able to demonstrate a clear correlation of RET expression with ER expression. Importantly, our data suggests that RET expression has no impact on overall survival and thus is not a prognostic factor in human breast cancer.

Since its initial discovery RET has increasingly gained importance in multiple cancer types including breast cancer [12, 26–31]. While RET expression in pancreatic cancer has been consistently reported to correlate with poor survival [26–28] results regarding survival in breast cancer are not entirely consistent [16] and only a few reports using human tissue exist. Gatelli et al. [16] examined RET expression in 89 breast cancer patients. They could demonstrate a correlation of high RET expression with larger tumour size as well as with a decreased metastasis-free and overall survival. Morandi et al. [19] analysed the RET co-receptor ligand GDNF and its signalling pathway in a GDNF-response gene set and correlated it to a dataset of 81 ER positive breast cancer patients as well as 597 breast cancer samples from “The Cancer Genome Atlas” [32] Not only did they find a significant higher GDNF score in Luminal B breast cancer subtype in both data sets, but also a decreased distant metastasis free survival, relapse free survival and overall survival in this subtype [19]. Together with the fact that the Luminal B subtype is characterised by poorer prognosis within ER positive breast cancers [33], these results suggest a correlation of an activated GDNF signalling cascade resulting in RET activation. Concordantly, we found high RET expression rates in the Luminal B breast cancer subtypes. Another study by Griseri et al. [34] performed a *RET* genotyping association study in a cohort of 93 ER positive breast cancers and also found a statistically significant association of RET over-expression with poor prognosis.

While our data did not confirm an impact of RET expression on prognosis in ER positive breast cancer, we were able to reproduce the association of RET and ER expression [4]. This correlation was underlined in the past by the discovery of a cross-talk of the RET and ER signalling cascade [6]. While *RET* and *GFRα* seem to be direct target genes for oestrogen signalling, RET signalling in turn increases ER independent transcriptional activation of ER*α* target genes [6]. Further exploration revealed that RET plays a significant role in the resistance of cancer cells to endocrine treatment [1]. By expressing RET, the tumour cells develop de novo resistance and become resistant after an initial response [7, 14, 15]. Plaza-Menacho et al. [14] have shown that *RET* downregulation results in a 6.2-fold increase in sensitivity of the ER positive MCF7 cell line to the antiproliferative effect of the selective ER modulator Tamoxifen. Moreover, GDNF stimulation caused resistance to the drug and targeting RET restored Tamoxifen resistance [14]. A consecutive TMA study of 245 primary human breast cancers showed an association between RET expression and recurrent disease after adjuvant Tamoxifen treatment [14].

In summary, the crosstalk between RET and ER is important in the development of endocrine resistance and specifically targeting RET within combination treatment regimens might help to restore endocrine resistance in ER positive tumours. Since a majority (70%) of breast cancers are ER positive [35], strategies are needed to address this therapeutic problem [7, 36].

While specific RET inhibitors are only beginning to emerge, two different types of inhibitory agents are currently evaluated within preclinical and clinical trials in breast cancer [1]. Small molecule tyrosine kinase inhibitors are therapeutic substances, which are not exclusively targeting RET but also other tyrosine kinases [37]. Some of them at least partially managed to overcome endocrine resistance in breast cancer cell lines and mouse models [15, 18–20]. However, clinical Phase I and II studies struggle with a lack of benefit or toxicity when using such inhibitors, for example Sorafenib, Imatinib or Sunitinib, as monotherapies [38–41]. Combination therapy of Sorafenib with endocrine therapy as well as Sunitinib with chemotherapy showed more promising results but further data is needed [42]. Vadentanib monotherapy as well as its use within combination schemes was well tolerated but failed to demonstrate improved survival [43]. Several clinical trials are ongoing [1]. However, none of these drugs have been approved for a cohort with an actionable alteration involving RET [44], stressing the importance of defining patients who might benefit from RET inhibition.

Recent studies identified more specific and potent RET inhibitors such as Sitravatinib, which are currently evaluated in thyroid and non-small cell lung cancer [44, 45]. These trials will hopefully enlighten the debate about their potential benefits and show a reduced toxicity.

While the results of small molecule tyrosine inhibitors are difficult to interpret, inhibition of RET downstream signalling elements, shared with other signalling pathways [14], seem to be currently more promising. The ER receptor contains two transcription active domains, activation function (AF)-1 and AF-2 [7]. While AF-2 activity is dependent on estrogen binding, AF-1 is regulated by phosphorylation mediated via extracellular signal–regulated kinases (ERK) 1/2, phosphatidylinositol 3-kinase (PI3K)/AKT, Protein kinase cAMP-dependent (PKA), cyclin A/E-CDK2, P21-Activated Kinase (PAK)1, cyclin-dependent kinase (CDK)7/Transcription factor II Human (TFIIH), p90RSK or p38 pathways. These are activated themselves by a large number of receptor tyrosine kinases including several growth factor receptor families [1, 7]. The activation of RET by GDNF in this context, has been demonstrated to increase ER*α* phosphorylation and estrogen-independent transcriptional activation of ER*α* target genes [14]. Interestingly, the RET downstream signalling in GDNF-treated MCF7 cells happens to a higher extent via the mechanistic target of Rapamycin (mTOR)/p70S6K pathway than the ERK1/2 and PI3K/AKT pathway [14]. This is confirmed by the fact, that RET downstream signalling was blocked with mTOR inhibitor Rapamycin, while a chemical inhibition of AKT and ERK1/2 had no impact [14]. By adding the mTOR inhibitor Everolimus to an aromatase inhibitor the BOLERO2 trial, managed to show an improved median progression-free survival of 6 months in ER positive breast cancer [46]. Therefore, current evidence suggests downstream elements of RET as possible therapeutic targets.

A general limitation of our study is that older samples might have suffered from loss of protein antigenicity, which might have affected the immunohistochemical staining outcome. A further point which needs to be mentioned is the method of defining the molecular subtypes. We used the definition of the St. Gallen Consensus Conference, which only provide an approximate definition of intrinsic subtypes and might lead to a underestimation of the impact of RET in the triple negative subtype [25, 47]. Another limiting factor is related to the follow-up in this study. Luminal A subtypes and T1/2 N0 stage cancers have a less aggressive course of disease and might recur even after 10 years. These late recurrences might therefore not be represented in our study due to the mean follow up period of 6.7 years.

A final limiting fact is the long recruitment period of 31 years which might have affected definitions of histopathological grading and staging. The same accounts for changing treatment regimens over time, which could have influenced the survival data.

## Conclusion

In summary, the results of our study confirm current knowledge that RET is an important element in ER positive breast cancer. However, our results, derived from a large breast cancer patient cohort with annotated clinicopathological data, underline the uncertainty regarding the prognostic impact of RET expression on patient survival in breast cancer and stresses the importance of further research. In light of this, further clinical trials identifying an adequate patient subgroup which might profit from RET inhibition as well as further studies investigating the impact of RET expression on patient outcome in breast cancer are clearly needed.

## Abbreviations

AF: Actviation Function
AJCC: American Joint Committee on Cancer
CDK: Cyclin-dependent kinase
EKNZ: Ethikkomission Nordwest- und Zentralschweiz
ER: Estrogen Receptor
ERK: Extracellular signal–regulated kinases
GDNF: E glial cell line-derived neurotrophic factor
GFRα1-4: GDNF family α-receptors
HER2: Human Epidermal Growth Factor Receptor 2
mTOR: Mechanistic Target of Rapamycin
PAK: P21-Activated Kinase
PIK3: Phosphatidylinositol 3-kinase
PKA: Protein kinase cAMP-dependent
PR: Progesterone Receptor
REMARK: Reporting recommendations for tumourmarker prognostic studies
RET: Rearranged during Transfection
RTK: Receptor tyrosine kinase
SD: Standard Deviation
TFIIH: Transcription factor II Human
TMA: Tissue Microarry
